# Regulation of Nuclear Factor κB (NF-κB) Transcriptional Activity via p65 Acetylation by the Chaperonin Containing TCP1 (CCT)

**DOI:** 10.1371/journal.pone.0042020

**Published:** 2012-07-31

**Authors:** Nadja Pejanovic, Karin Hochrainer, Tao Liu, Birgit L. Aerne, Miguel P. Soares, Josef Anrather

**Affiliations:** 1 Division of Neurobiology, Department of Neurology and Neuroscience, Weill Cornell Medical College, New York, New York, United States of America; 2 Instituto Gulbenkian de Ciência, Oeiras, Portugal; 3 Medical Research Council (MRC) Laboratory for Molecular Cell Biology, University College London, London, United Kingdom; 4 Apoptosis and Proliferation Control Laboratory, Cancer Research UK, London Research Institute, London, United Kingdom; Chang Gung University, Taiwan

## Abstract

The NF-κB family member p65 is central to inflammation and immunity. The purpose of this study was to identify and characterize evolutionary conserved genes modulating p65 transcriptional activity. Using an RNAi screening approach, we identified chaperonin containing TCP1 subunit η (CCTη) as a regulator of *Drosophila* NF-κB proteins, Dorsal and Dorsal-related immunity factor (Dif). CCTη was also found to regulate NF-κB-driven transcription in mammalian cells, acting in a promoter-specific context, downstream of IκB kinase (IKK). CCTη knockdown repressed *IκBα* and *CXCL2/MIP2* transcription during the early phase of NF-κB activation while impairing the termination of *CCL5/RANTES* and *CXCL10/IP10* transcription. The latter effect was associated with increased DNA binding and reduced p65 acetylation, presumably by altering the activity of histone acetyltransferase CREB-binding protein (CBP). We identified p65 lysines (K) 122 and 123 as target residues mediating the CCTη-driven termination of NF-κB-dependent transcription. We propose that CCTη regulates NF-κB activity in a manner that resolves inflammation.

## Introduction

Nuclear factor κB (NF-κB) proteins comprise a family of eukaryotic transcription factors that control the expression of a large number of genes regulating inflammation and immunity as well as developmental processes including cellular growth and apoptosis [1]. Unfettered NF-κB activation has been associated with the pathogenesis of a number of inflammatory diseases [2]. In their active form, NF-κB proteins are nuclear homo- or hetero-dimeric complexes composed of p65/RelA, RelB, cRel, p105/p50, and p100/p52. The prototypical and most ubiquitously expressed NF-κB dimer is composed of p50 and p65 subunits where p65 is the main transcriptional activator [3].

Under homeostatic conditions, NF-κB activity is constitutively repressed by its interaction with cytoplasmic NF-κB inhibitors (IκB) [4]. While inducible degradation of IκB molecules is a central mechanism regulating p65 transcriptional activity, posttranslational modifications are also important for its activity [5], [6]. These include phosphorylation, which modulates DNA binding, interactions with other proteins as well as p65 stability [5]. Phosphorylation often precedes other posttranslational modifications such as acetylation and ubiquitination [7]–[10]. For example, p65 phosphorylation at serine (S) 276 facilitates interaction with CREB-binding protein (CBP)/p300 and diminishes histone deacetylase 1 (HDAC1) binding, leading to p65 acetylation [11].

Lysine (K) acetylation is mostly a nuclear event [6], controlling p65 transcriptional activity [12]–[15] as well as the duration of NF-κB activation via regulation of DNA binding [13], [16] and association with IκBα [13]. Specific p65 residues may be preferentially targeted by different histone acetyltransferases (HAT), which include CBP, p300 and p300/CBP-associated factor (P/CAF) [13], [14], [16]. The acetylation status of p65 is controlled by the opposing activities of HATs and HDACs including HDAC1 [17], HDAC3 [12], [16], SIRT1 [18], and SIRT2 [19]. Beside acetylation, p65 lysine residues can be modified by methylation and ubiquitination resulting in altered transcriptional activity or proteasomal degradation [20]–[24].

NF-κB proteins and the signal transduction pathways leading to their activation are highly evolutionary conserved. As in mammals, *Drosophila* p65 homologues Dorsal and Dif are constitutively inhibited by the IκB like molecule Cactus [25], [26]. Toll receptor activation leads to Cactus degradation, Dorsal and Dif nuclear translocation and transcription of NF-κB dependent genes such as *Drosomycin* and *Cecropin* [27]–[29]. Little is known about the impact of posttranslational modifications on Dorsal and Dif transcriptional activity. Yet, several phospho-acceptors as well as lysine residues targeted by posttranslational modifications in p65 are conserved in Dorsal and Dif. Supporting this view, phosphorylation of Dorsal S312 and S317, which correspond to p65 S276 and S281, can control Dorsal nuclear translocation [30], [31].

We set out to identify evolutionary conserved genes regulating NF-κB-dependent gene transcription using a functional RNAi based gene screen in *Drosophila* cells. We reveal the chaperonin containing TCP1 subunit eta (CCTη) as a novel gene regulating NF-κB acetylation and activity.

## Materials and Methods

### Cell Culture

*Drosophila* S2 cells were kindly provided by Monica Bettencourt Dias (Instituto Gulbenkian de Ciência, Oeiras, Portugal) [32] and were grown at 25°C in Schneider’s Drosophila medium (Invitrogen). HeLa and HEK293 cells were obtained from ATCC. Mouse embryonic fibroblasts (MEF) isolated from *Rela* ^−/−^ mice were kindly provided by Dr. Amer Beg (Moffitt Cancer Center, Tampa, FL) and have been previously described [33]. *Rela* ^−/−^ MEF were retrovirally transduced with human RelA wt, K122/123R, K221R or K310R mutants essentially as described [34]. Cells were grown at 37°C in DMEM (MediaTech) supplemented with 10% FBS, 100 units/ml penicillin G and 100 µg/ml streptomycin B (all Atlanta Biologicals) in a humidified atmosphere containing 5% CO_2_.

### Plasmid Constructs

Drosomycin-luciferase (*Drs-luc*) and Toll 10b expression vectors were kindly provided by Dr. Mika Ramet (University of Tampere, Tampere, Finland) and were described elsewhere [35]. Dorsal and Dif were amplified from the original pAct-dl and pAct-Dif vectors (kind gift from Dr. Ylva Engstrom, Stockholm University, Stockholm, Sweden [29]) by PCR (Dorsal 5′-GAT CCT CGA GAT GTT TCC GAA CCA GAA CAA TGG AG-3′ and 5′-TCG ATC TAG ACG TGG ATA TGG ACA GGT TC-3′; Dif 5′-GAT CCT CGA GAT GTT TGA GGA GGC TTT C-3′ and 5′-TCG ATC TAG ATT TGA ATG GCT GAA TTC CCA AG-3′) and cloned (*Eco*RV/*Xba*I) and (*Xho*I/*Xba*I) into pAc5.1/V5-HisA vector (Invitrogen), respectively. NF-κB luciferase reporter construct, i.e. *κB-luc*, has been described elsewhere [36]. Scrambled, CCTη, CCTα and CCTζ shRNA constructs were cloned by inserting annealed oligonucleotides containing a non-targeting (5′-GAG TGT TTG AGT TTG AGA TCC-3′) or a unique 19–21 bp sequence derived from the mRNA transcript of CCTη (5′-GCC ACA AAC ATT CTC AAC A-3′), CCTα (5′-AAA TAC TAA GGC TCG TAC GTC-3′) or CCTζ (5′-AAG TCT GTG GCG ATT CAG ATA-3′) gene between the unique *Bgl*II and *Hind*III restriction sites of the pSUPER vector (OligoEngine). The N-terminal myc-tagged p65 expression vector has been described [37]. C-terminal HA-tagged CBP was expressed from a pcDNA3 vector. p65 K122/123R, K221R and K310R expression vectors were generated by site-directed mutagenesis and cloned into the pLXIH vector as described [34]. All constructs were verified by automated DNA sequencing using dye termination chemistry.

### Transient Transfection and Reporter Assay

S2 cells were cultured in 12-well plates (5×10^5^ cells per well) and a day after exposed to 1 µg of total DNA (150 ng *Drs-luc*, 5 ng Toll 10b, 50 ng Dorsal, 50 ng Dif; the total amount of DNA was kept constant by using empty vector) and 5 µl of Cellfectin transfection reagent according to manufacturer’s instructions (Invitrogen) in *Drosophila* SFM medium (Invitrogen) for 12 h at 25°C. Cells were washed and transferred into 96-well plates containing lacZ dsRNA (900ng per 5×10^4^ cells). After incubation for three days, firefly (Luc) and Renilla (Ren) luciferase activity were measured using Dual-luciferase Reporter Assay System (Promega). HEK293 cells were grown in 12-well plates and transfected at 90% confluency. Cells were exposed to 1 µg of total DNA (300 ng of reporter plasmid, 500 ng of shRNA plasmid and 40 ng of CMV enhancer/β-gal control plasmid; the total amount of DNA was kept constant by using empty vector) and 1 µl of Lipofectamine 2000 reagent according to manufacturer’s instructions (Invitrogen) in DMEM for 3 h. After addition of FBS to a final concentration of 10%, cells were incubated (three days, 37°C), stimulated with TNF (10 ng/ml, 6 h), lysed with passive lysis buffer (Promega) and supernatants were assayed for luciferase and β-gal activity as described elsewhere [36].

### RNA Interference, Transient Transfection and Reporter Assay

S2 cells were cultured in 12-well plates and transfected as above (150 ng *Drs-luc* reporter plasmid, 5 ng Toll 10b plasmid; the total amount of DNA was kept constant by using empty vector). Cells were washed, diluted to 1×10^6^ cells/ml and a total of 10 µl of cells were added to the dsRNA-containing 384-well plate (250 ng dsRNA/well), resulting in a final concentration of 1×10^4^ cells per well. Plates were gently centrifuged, cells were incubated (30 min, room temperature) and 30 µl of Schneider’s medium containing 15% FBS was added. Plates were sealed to prevent evaporation and incubated for three days at 25°C. Luciferase (Luc) and Renilla (Ren) activity was measured using Dual-luciferase Reporter Assay System according to manufacturer’s instruction (Promega). 89 wells not containing dsRNA on each experimental plate were used to calculate the average Luc and Ren activities. The positive control in screen and data processing, dsRNA of DIAP1 inhibitor of apoptosis, led to a very significant reduction of Luc and Ren signals in two out of three experimental plates that were considered for further analysis. Experimental data was normalized by setting the average Luc and Ren values of wells not containing dsRNA to 1. dsRNA treatments that modulated Luc expression (values lying outside the mean ±2SD boundaries) with no effect on Ren values (values lying within the mean ± SD boundaries) in two experiments were considered indicative for putative candidate genes.

### dsRNA Synthesis and Screen Confirmation

dsRNAs targeting putative genes identified in the screen were generated by *in vitro* transcription of a PCR-generated DNA template (RNAi probes, FlyBase) containing the T7 promoter sequence on both ends (RiboMAXTM Large Scale RNA Production System-T7, Promega) and purified using Mini Quick Spin RNA Columns (Roche). Transiently transfected S2 cells (150 ng *Drs-luc*, 50 ng Dorsal, 50 ng Dif, 5 ng Toll 10b per 5×10^5^ cells) were exposed to dsRNA (900 ng per 5×10^4^ cells) in 96-well plate, incubated for three days and Luc and Ren activity were measured.

### CCTη Knockdown by Small Interfering RNA (siRNA)

siRNA-mediated knockdown of CCTη was performed in a 12-well plate format in HeLa (6×10^4^ cells per well), HEK293 (3×10^5^ cells per well) and MEF (5×10^4^ cells per well) cells. Cells were exposed to 50 nM siGENOME Non-Targeting siRNA #1 or CCTη siRNA and 1 µl/ml of DharmaFECT1 transfection reagent according to manufacturer’s instructions (Thermo Scientific). CCTη siRNA target sequences were human: 5′-GCC ACA AAC ATT CTC AAC A-3′ and mouse: 5′- GCC ACA AAC ATC CTC AAC A -3′.

### Gene Expression Analysis

Real-time quantitative PCR (qPCR) was carried out using SYBR Green chemistry (Invitrogen) on a Chromo4 continuous fluorescence monitoring thermocycler (MJ Research) as described previously [34]. Relative transcript levels were determined by normalization to the housekeeping gene *HPRT*. Primers used for expression analysis in HeLa cells were: *IκBα* (5′-TCC TGT TGA AGT GTG GGG CTG ATG-3′ and 5′-CCT CCA AAC ACA CAG TCA TCA T-3′), *CXCL2* (5′-CAC TCA AGA ATG GGC AGA AAG-3′ and 5′-TCA GGA ACA GCC ACC AAT AAG-3′), *IL8* (5′-TCC TGA TTT CTG CAG CTC TGT-3′ and 5′- TGT GGT CCA CTC TCA ATC ACT C-3′), *TNF* (5′-AGT GCT GGC AAC CAC TAA GAA-3′ and 5′-ATT CCA GAT GTC AGG GAT CAA-3′), *CXCL10* (5′-CCT CTC CCA TCA CTT CCC TAC-3′ and 5′-GCT GAT TTG GTG ACC ATC ATT-3′), *CCL5* (5′-TGC CCA CAT CAA GGA GTA TTT-3′ and 5′-CCA TCC TAG CTC ATC TCC AAA-3′) and *HPRT* (5′-TTC TGT GGC CAT CTG CTT AGT-3′ and 5′-GCC CAA AGG GAA CTG ATA GTC-3′). Primers used for expression analysis in MEF were: *Cxcl10* (5′-AAG TCA GCC AAT CAG GAC TCA-3′ and 5′-GTT GGC TCG GGA TGT CTC T-3′). Primers for the mouse *Hprt* gene have been described elsewhere [34]. Expression levels of unstimulated, scrambled siRNA-exposed cells were set to 1, and fold induction for other experimental groups was calculated. *CXCL10* mRNA levels of TNF-unstimulated Hela cells were not consistently detectable; expression level of *CXCL10* mRNA in CCTη siRNA-exposed cells 16 h after TNF was set to 100 and relative values for other experimental groups were calculated.

### Preparation of Subcellular Extracts, Electrophoretic Mobility Shift Assay (EMSA) and Western Blotting

HeLa cells were transfected with siRNA as described above and incubated for three days before stimulation with TNF (10 ng/ml). Preparation of cytoplasmic/nuclear extracts and EMSA were carried out as described previously [37]. Briefly, cells were lysed in hypotonic buffer (20 mM HEPES pH 7.9, 1.5 mM MgCl_2_, 10 mM KCl, 0.5% TritonX-100, supplemented with protease inhibitors) and centrifuged (600 *g*, 5 min, 4°C). Supernatant (cytoplasmic fraction) was transferred into a new tube and the remaining pellet (nuclear fraction) was washed once in hypotonic buffer, lysed (20 mM HEPES pH 7.9, 25% glycerol, 1.5 mM MgCl_2_, 400 mM NaCl, 0.2 mM EDTA, 1 mM PMSF, 1 mM DTT, protease inhibitors), and cleared by centrifugation. Nuclear extracts were incubated with 100,000 cpm of double-stranded [γ-^32^P]ATP-radiolabled NF-κB oligonucleotide (5′-AGT TGA GGG ACT TTC CCA GGC-3′; 30 min, room temperature; PerkinElmer) and the resulting DNA-protein complexes were separated on a 6% polyacrylamide gel in Tris/glycine/EDTA buffer pH 8.5. Experimental data were analyzed by setting the net intensity of EMSA bands to 100 and calculating the percentage of intensity for each data point. For supershift analysis, labeled DNA-protein complexes were incubated with 1 µg of p65 (sc-8008x, Santa Cruz) or p50 (sc-114x, Santa Cruz) specific antibodies or non-immune rabbit IgG (I5006, Sigma) (1 h, 4°C) prior to separation. For western blotting, proteins contained in cytoplasmic and nuclear fractions were resolved by electrophoresis on 10% SDS-polyacrylamide gels, transferred to PVDF membranes and detected with antibodies against IκBα (IMG-127A, Imgenex), p65 (sc-372, Santa Cruz), p50 (sc-7178, Santa Cruz), CCTη (sc-13889, Santa Cruz), GAPDH (MAB374, Millipore) and Sp1 (sc-59, Santa Cruz). Primary antibodies were detected using HRP-conjugated secondary antibodies (Santa Cruz).

### Immunoprecipitation and Protein Acetylation

HEK293 cells were grown in 12-well plates and transfected with calcium phosphate. Cells were exposed to 1.6 µg of total DNA (50 ng of myc-tagged p65, 200 ng of HA-tagged CBP expressing construct and 1 µg of shRNA plasmid; the total amount of DNA was kept constant by using empty vector), incubated for three days and stimulated with TNF (10 ng/ml, 30 min). Cells were lysed (50 mM HEPES pH 7.9, 250 mM NaCl, 1% NP-40, 1 mM EDTA, protease inhibitors) and centrifuged (10 min, 16000 *g*, 4°C). Supernatants were incubated with 15 µl c-myc-conjugated agarose beads (2 h, 4°C, Sigma). For CBP detection, cells were transfected with CCTη or scrambled shRNA plasmid, stimulated with TNF (10 ng/ml, 30 min) and cell extracts were incubated with 1 µg CBP antibody (o/n, 4°C, sc-369, Santa Cruz) and 20 µl Protein-A Sepharose beads (1 h, 4°C, GE Healthcare). For CCTη detection, HEK293 cells were exposed or not to CCTη or scrambled siRNA and stimulated with TNF (10 ng/ml) for indicated times. Nuclear extracts were incubated with 1 µg TCP-1η antibody (sc-13889, Santa Cruz) and 20 µl Protein-A Sepharose beads. Precipitated proteins were washed in lysis buffer, resuspended in 2x SDS sample buffer, resolved by SDS-PAGE and subjected to western blotting with antibodies detecting acetylated lysine (9814S, Cell Signaling), p65 (sc-372, Santa Cruz), CBP or CCTη Primary antibodies were detected using Protein-A-HRP conjugates (Millipore) or respective secondary antibodies. Purity of nuclear extracts was confirmed using anti-GAPDH antibody (MAB374, Millipore).

## Results

### Inhibition of CCTη Expression Modulates NF-κB-Dependent Reporter Activity

**Figure 1:**
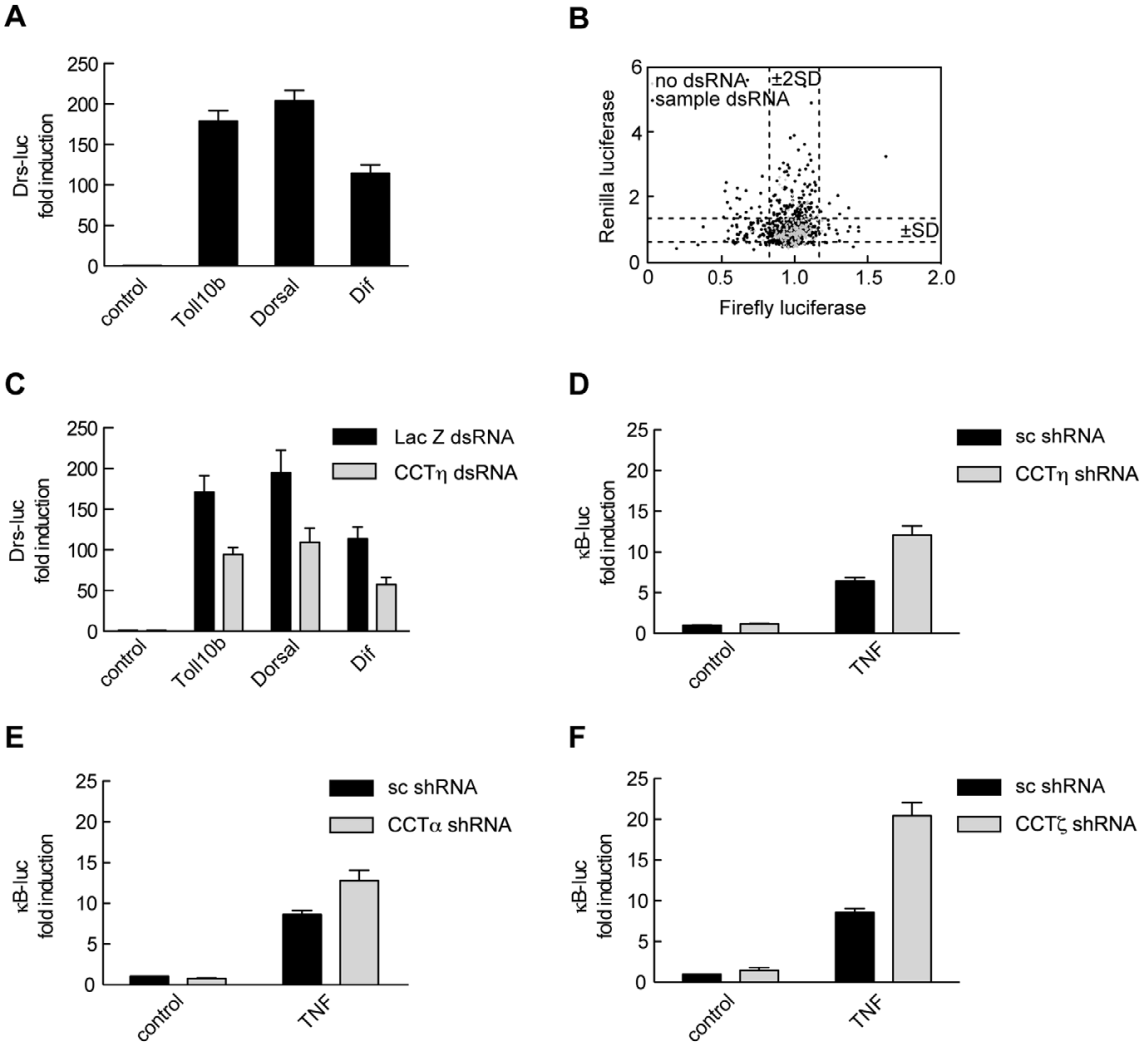
Identification of CCTη as a novel gene regulating NF-κB transcriptional activity. **A:** S2 cells were transiently transfected with the *Drs-luc* reporter plus or minus Dorsal, Dif or Toll 10b and exposed to LacZ dsRNA. Illustrated data represent mean firefly luciferase normalized to Renilla luciferase units ± SEM (n≥11 derived from four independent experiments). **B:** S2 cells were transiently transfected with the *Drs-luc* reporter plus Toll 10b and seeded into wells of a 384-well plate containing 265 *Drosophila* kinases and kinase regulatory subunits. Firefly and Renilla luciferase values from two independent experimental plates were plotted. Control wells not containing dsRNA are illustrated with the light gray diamonds. **C:** S2 cells were transiently transfected as in **A** and exposed to CCTη or LacZ dsRNA. Illustrated data represent mean firefly luciferase normalized to Renilla luciferase units ± SEM (n = 9, derived from three independent experiments). **D**–**F:** HEK293 cells were transiently transfected with κB-luc reporter together with CCTη, CCTα, CCTζ or scrambled (sc) shRNA, incubated for three days and stimulated with TNF (10 ng/ml, 6 h) or left untreated (control). Results shown are mean normalized firefly luciferase units ± SEM (n≥7, derived from three independent experiments).

To identify evolutionary conserved genes regulating the NF-κB pathway we employed a dsRNA library targeting 265 putative *Drosophila* kinases and kinase regulatory proteins. *Drosomycin*-driven *luciferase* (*Drs-luc*) expression can be induced by a constitutively active form of Toll (Toll 10b) as well as by *Drosophila* p65 homologues Dorsal and Dif [35], [38]. *Drs-luc* expression increased significantly in *Drosophila* S2 cells co-transfected with Toll 10b, Dorsal or Dif, as compared to cells transfected with *Drs-luc* alone (Fig. 1A). We used the same assay to perform an RNAi based gene screen in S2 cells transiently transfected with *Drs-luc* reporter, Toll 10b expression construct and a constitutively active *pAct5-Renilla* reporter. Firefly and Renilla luciferase values were plotted and dsRNA treatments that significantly modulated firefly luciferase expression (values lying outside the mean ±2SD) with no effect on Renilla luciferase values were further validated (Fig. 1B). We identified chaperonin containing Tcp-1 subunit η (CCTη), an evolutionary conserved protein not previously implicated in the regulation of NF-κB signaling. We confirmed that CCTη regulates NF-κB activity in a reporter assay in S2 cells. CCTη dsRNA inhibited Toll 10b-, Dorsal- and Dif-induced *Drs-luc* expression, as compared to control LacZ dsRNA (Fig. 1C).

To investigate whether CCTη controls mammalian NF-κB signal transduction pathway we assessed its effect on TNF-induced NF-κB reporter activity. HEK293 cells were transiently transfected with a *κB-luc* reporter and CCTη shRNA or scrambled shRNA as a control. In contrast to S2 cells, CCTη knockdown in mammalian cells enhanced TNF-driven reporter activity, as compared to scrambled shRNA transfected controls (Fig. 1D). The same result was observed when two other CCT subunits, CCTα and CCTζ, were targeted by shRNA (Fig. 1E, 1F), suggesting the involvement of the functional chaperonin CCT complex in the regulation of NF-κB activity in mammalian cells.

### CCTη Modulates NF-κB-Dependent Gene Expression

**Figure 2:**
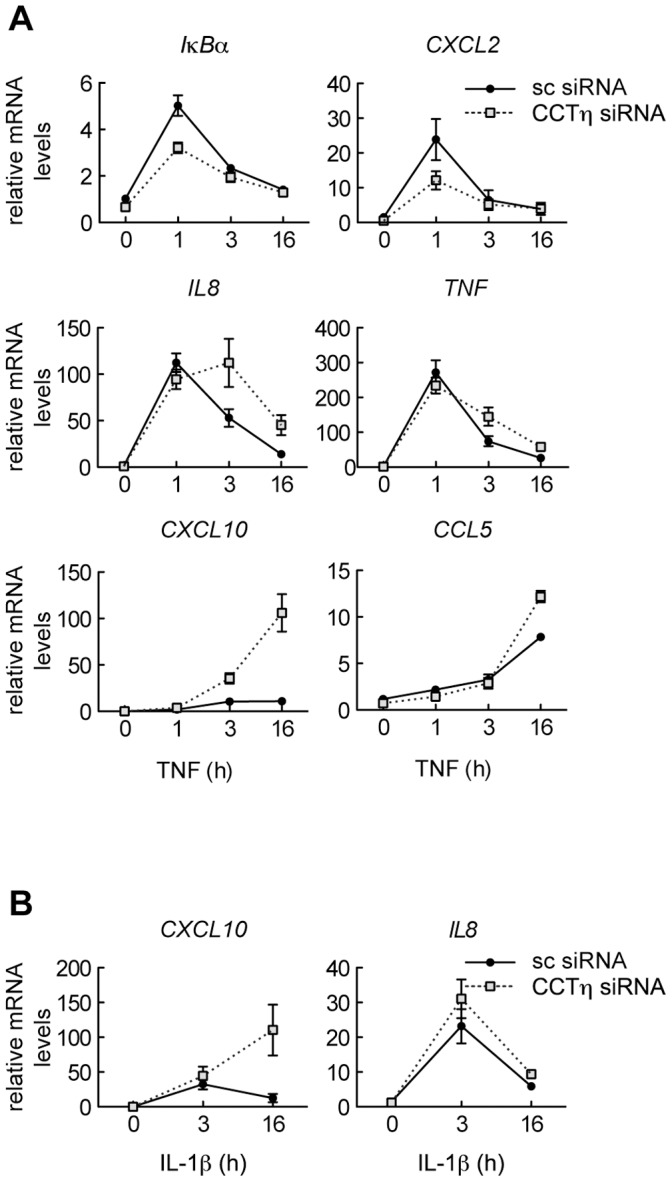
CCTη modulates TNF and IL-1β-induced gene expression in a promoter specific way. HeLa cells were exposed to CCTη or scrambled siRNA for three days and stimulated with **A:** TNF (10 ng/ml) or **B:** IL-1β (10 ng/ml) for indicated times. mRNA levels were analyzed by qPCR. Data represent mean relative mRNA levels ± SEM (n≥3).

The canonical NF-κB signal transduction pathway can be activated via different receptors, including TNF and IL-1 receptors, that initiate distinct signaling cascades converging at the level of the IκB kinase (IKK) complex [39]. To assess whether CCTη regulates both pathways we analyzed mRNA expression of NF-κB target genes by qPCR in HeLa cells transfected with CCTη or scrambled siRNA and stimulated with either TNF or IL-1β. We found that CCTη affects NF-κB-dependent gene expression in a promoter-specific context. Whereas *IκBα* and *CXCL2* mRNA expression was reduced at 1 h after TNF stimulation in CCTη siRNA transfected cells, expression of *TNF*, *IL-8*, *CXCL10* and *CCL5* mRNA increased at 3 and/or 16h after TNF stimulation, as compared to scrambled siRNA transfected controls (Fig. 2A). Similarly to TNF stimulation, CCTη knockdown led to increased *IL-8* and *CXCL10* mRNA expression 3 and 16 h after IL-1β addition (Fig. 2B). This suggests the involvement of CCTη in initiation as well as termination of NF-κB-driven transcription, revealing a multi-faced role in regulating NF-κB-dependent genes, possibly acting downstream of IKKs.

### CCTη Regulates NF-κB DNA Binding

**Figure 3:**
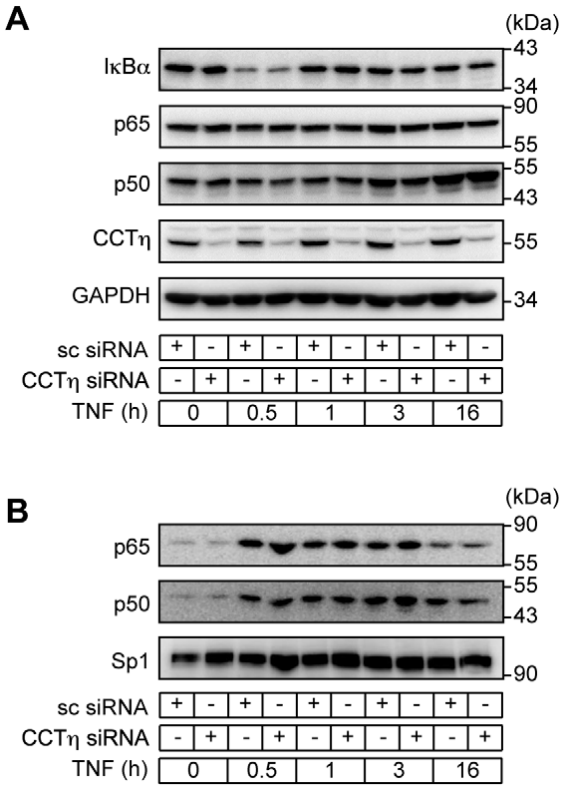
CCTη does not influence NF-κB subcellular distribution. HeLa cells were exposed to CCTη or scrambled (sc) siRNA for three days and stimulated with TNF (10 ng/ml) for indicated times. **A:** IκBα, p65, p50, CCTη and GAPDH were detected in cytoplasmic extracts and **B:** p65, p50 and Sp1 were detected in nuclear extracts by western blotting. Immunoblots are representative of three independent experiments.

**Figure 4:**
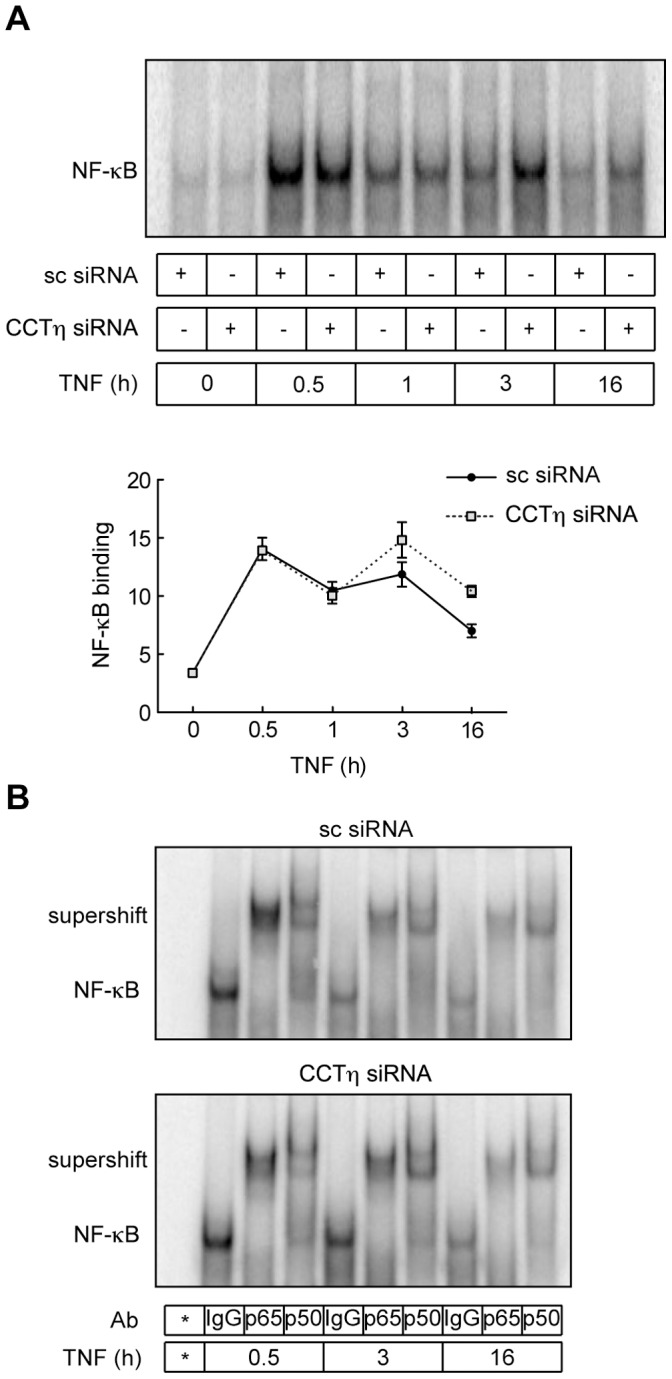
CCTη knockdown increases NF-κB DNA binding. HeLa cells were exposed to CCTη or scrambled (sc) siRNA for three days and stimulated with TNF (10 ng/ml) for indicated times. **A:** Nuclear extracts were analyzed by EMSA using a κB consensus dsDNA oligonucleotide. Data shown represent mean intensity ± SEM (n = 3). **B:** Supershift analysis of NF-κB complexes composed of p65/p50 hetero-dimers representative of two independent experiments. Ab, antibody; *, free probe.

Proteolytic IκB degradation is required for NF-κB nuclear translocation, binding to the κB enhancer and initiation of transcription [5]. In addition, NF-κB-dependent IκBα re-synthesis is critical for termination of NF-κB signaling pathway [40], [41]. Thus, we investigated whether CCTη modulates IκBα protein levels. CCTη knockdown had no apparent impact on TNF-induced IκBα degradation, as compared to scrambled siRNA transfected cells (Fig. 3A). In accordance, cytoplasmic and nuclear p65 and p50 protein levels in TNF-treated cells were not influenced by CCTη siRNA transfection (Fig. 3A, 3B), suggesting that CCTη exerts its effect independently of IκBα, presumably after p65 nuclear translocation. Indeed, we found that CCTη knockdown was associated with increased NF-κB binding to DNA κB consensus sequence at 3 and 16 h after TNF stimulation, while not affecting binding at 30 min and 1 h, as analyzed by EMSA (Fig. 4A). Resolved DNA protein complexes were identified as p50/p65 heterodimers by specific binding of these complexes to both p50 and p65 specific antibodies (Fig. 4B). Composition of NF-κB heterodimers was not changed at different time points after TNF stimulation or by CCTη knockdown (Fig. 4B), indicating that CCTη does not affect NF-κB dimer formation. This suggests that increased DNA binding at 3 and 16 h after TNF stimulation could account for increased expression of NF-κB target genes at these time points in CCTη siRNA transfected cells. Impaired gene expression at 1 h after TNF, on the other hand, cannot be explained by altered DNA binding. Several post-translational modifications that have been shown to modulate NF-κB transcriptional activity without affecting its DNA binding [6] could account for this effect.

### CCTη Modulates p65 Transcriptional Activity via a Mechanism that Targets K122/123 Acetylation

**Figure 5:**
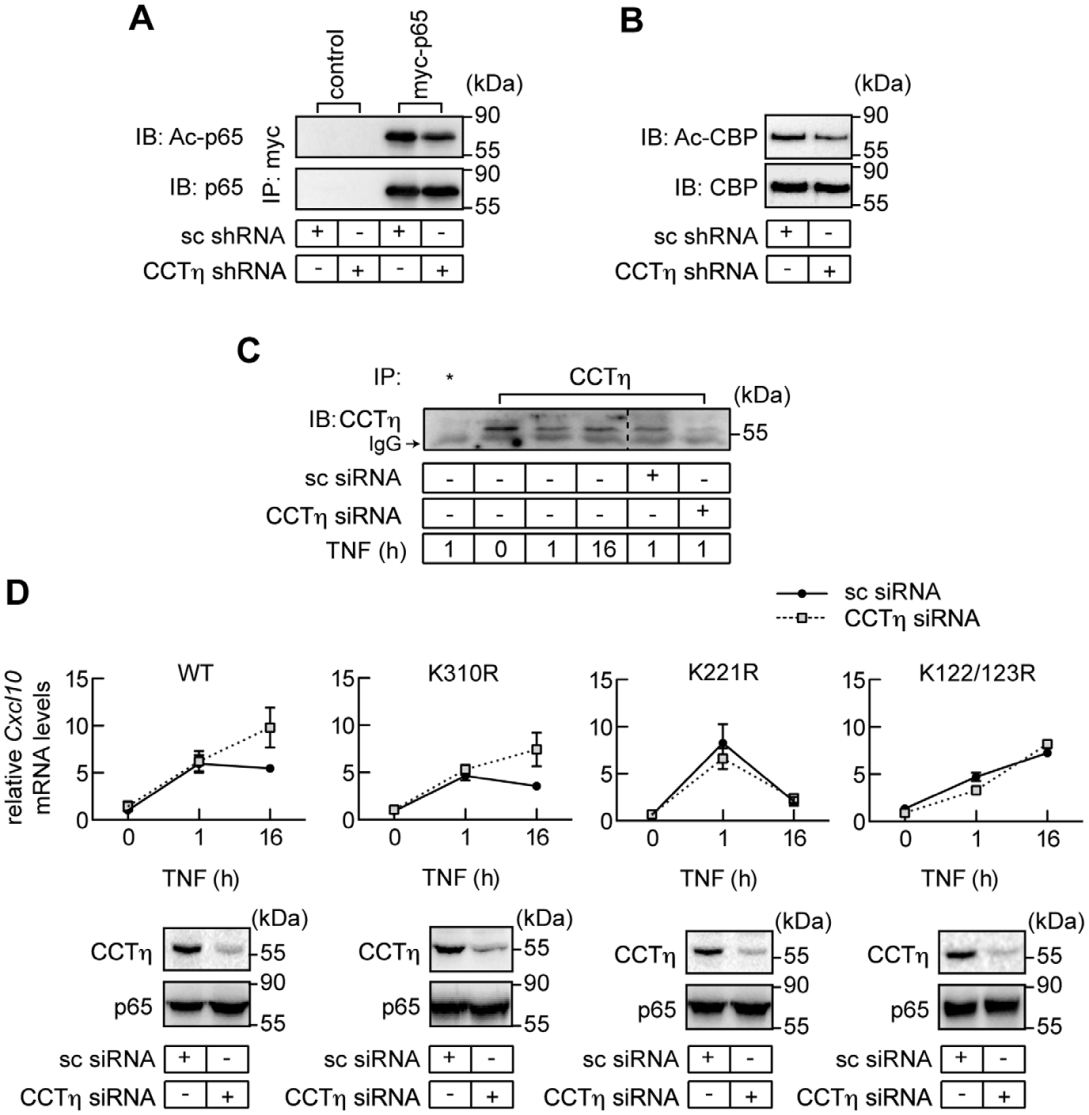
CCTη regulates p65 transcriptional activity by modulating CBP-dependent p65 acetylation of K122 and K123. **A:** HEK293 cells were transfected with CBP, pCDNA3 (control) or myc-p65 and scrambled (sc) or CCTη shRNA, incubated for 3 days and p65 was immunoprecipitated using c-myc-specific agarose beads. Acetylated p65 (Ac-p65) and total p65 were detected using antibodies to acetylated lysine or p65, respectively. **B:** HEK293 cells were transiently transfected with scrambled (sc) or CCTη shRNA, incubated for three days and endogenous acetylated CBP (Ac-CBP) and total CBP were detected using antibodies to acetylated lysine or CBP after immunoprecipitation, respectively. **C:** HEK293 cells were transfected or not with scrambled (sc) or CCTη siRNA, stimulated with TNF (10ng/ml) for indicated times and nuclear extracts were prepared. CCTη was immunoprecipitated using TCP-1η antibody. Unrelated EP-1 antibody was used as a goat IgG control (*). **D:** *Rela* ^−/−^ MEF reconstituted with either WT p65, K310R, K221R or K122/123R mutant were exposed to scrambled (sc) or CCTη siRNA for three days and stimulated with TNF (10 ng/ml) for indicated times. *Cxcl10* mRNA levels were analyzed by qPCR as described under “Materials and Methods”. Data represent mean relative mRNA levels ± SEM (n≥3). CCTη and p65 were detected in cell extracts by western blotting (representative of three independent experiment).

CBP/p300 plays a central role in terminating nuclear NF-κB activity by affecting p65 transcriptional activity, IκBα interaction and DNA binding [13], [16]. HDAC3, on the other hand, can antagonize the effect of CBP/p300 by deacetylation of p65 [13], [16]. Given that the chaperonin CCT is required for HDAC3 activity [42], we hypothesized that CCTη regulates NF-κB DNA binding via modulation of HDAC3-dependent p65 deacetylation. If this were the case then knockdown of CCTη should increase p65 binding to DNA κB consensus sequence by increasing p65 acetylation [13]. To test this hypothesis HEK293 cells were transfected with myc-tagged p65, CBP-encoding expression vector and either CCTη- or non-targeting shRNA constructs. p65 acetylation was assessed after immunoprecipitation by immunoblotting with anti-acetyl lysine antibody. Contrary to our expectation, CCTη knockdown decreased CBP-induced p65 acetylation (Fig. 5A), ruling out HDAC3 as a target of the CCTη-mediated effect on NF-κB activity in this experimental system. We next tested whether reduced CBP activity could account for decreased p65 acetylation in CCTη-depleted cells. CBP activity is regulated by several posttranslational modifications and auto-acetylation has been shown to enhance its enzymatic activity [43]. Therefore, we assessed CBP acetylation status in CCTη-depleted cells. CCTη knockdown did not alter CBP protein levels, but reduced CBP acetylation when compared to scrambled shRNA transfected cells (Fig. 5B). This suggests that CCTη regulates NF-κB transcriptional activity through modulation of p65 acetylation by controlling CBP activity.

While CCT proteins are predominantly localized in the cytoplasm, their nuclear localization and function have recently been reported [44]–[47]. Since p65 acetylation is thought to be a nuclear event [6] and given that CBP/p300 is expressed in the nucleus [48]–[50] we assessed whether chaperonin CCTη was expressed in the nucleus. CCTη was detected in the nucleus of both non-stimulated and TNF stimulated HEK293 cells after immunoprecipitation (Fig. 5C). Specificity was confirmed by lack of nuclear CCTη in CCTη siRNA-transfected cells (Fig. 5C).

Since CBP/p300 can acetylate p65 on multiple lysines [13], [14], [16], we assessed whether the effect of CCTη on NF-κB-dependent gene regulation occurs through acetylation on specific p65 lysine residues. To this end, we compared TNF-induced *Cxcl10* mRNA expression in p65^−/−^ MEF retrovirally reconstituted with WT p65, K310R, K221R or K122/123R p65 mutants, exposed to scrambled or CCTη siRNA. All MEF cell lines showed comparable *Cxcl10* mRNA levels, as assessed 1 h after TNF stimulation (Fig. 5D). Analogous to results obtained in HeLa cells (Fig. 2A), CCTη knockdown in MEF expressing WT p65 increased *Cxcl10* expression at 16 h after TNF stimulation, as compared to scrambled siRNA transfected controls (Fig. 5D). Similarly to the WT p65, CCTη knockdown increased p65 K310R mutant activity at the later time point, suggesting that p65 acetylation at this K residue is not required for CCTη effect (Fig. 5D). K221 mutation abolished p65 transcriptional activity 16 h after TNF stimulation (Fig. 5D), possibly by enhancing IκBα binding and leading to p65 nuclear export, as previously reported [13]. Because the effect of CCTη on *Cxcl10* expression is only evident at the later phase of NF-κB- dependent transcription (Fig. 5D), we were not able to evaluate a possible effect of CCTη on this K mutant. CCTη knockdown failed to modulate the transcriptional activity of K122/123R p65 mutant (Fig. 5D), suggesting that CCTη facilitates p65 acetylation on lysines 122 and/or 123, reducing p65 DNA binding and gene transcription.

## Discussion

CCT is a ubiquitously expressed multimeric protein complex involved in protein folding and assembly of protein complexes in an ATP-dependent manner. Though originally described as a cytosolic protein involved in actin and tubulin folding [51]–[53], CCT is also expressed in the nucleus [44]–[47] (Fig. 5C). CCT can interact with a range of proteins, including Von Hippel-Lindau (VHL)-elongin BC tumor suppressor complex [54], cell-division cycle protein 20 (Cdc20) [55], sphingosine kinase 1 [56], Polo-like kinase 1 [57], HDAC3 [42], huntingtin [58], among others [59]. This suggests the involvement of CCT in various cellular functions beyond cytoskeleton organization, such as cell cycle, transcription, chromatin remodeling and protein degradation.

CCT is composed of eight different subunits (CCTα-θ) that share similar domains and amino acid sequences conserved across species [60]–[62]. Here we identify the chaperonin CCT subunit η (CCTη) as an evolutionary conserved regulator of NF-κB-dependent transcription that exhibits its effect via a mechanism that targets p65 acetylation. While we have focused on the CCTη subunit, it is likely that a functional chaperonin complex is needed to regulate NF-κB transcription. This notion is supported by the observation that knockdown of the CCT subunits α or ζ had comparable effects to that of CCTη in regulating TNF-induced reporter activity (Fig. 1E, 1F), consistent with the notion that depletion of individual CCT subunits reduces the expression and activity of the CCT chaperonin complex [63], [64].

We have established that CCT regulates NF-κB transcriptional activity, presumably at a nuclear level. CCT knockdown modulated both TNF- and IL-1β-driven NF-κB activation (Fig. 2), suggesting that it acts downstream of IKK activation, a common denominator of the signal transduction pathways triggered by cross-linking of the TNF and IL-1 receptors [39]. In addition, CCT knockdown failed to modulate IκBα degradation as well as p65 nuclear translocation (Fig. 3). Consecutively, we found that CCT knockdown increased NF-κB binding to DNA κB consensus sequence (Fig. 4), and we attribute this effect to altered p65 acetylation.

In *Drosophila* cells, CCT knockdown reduces NF-κB-driven reporter activity, which contrasts with mammalian cells in which CCT depletion promotes NF-κB activity in response to TNF (Fig. 1C–F). This apparent discrepancy could result from opposing functional outcome of NF-κB acetylation in *Drosophila vs.* mammalian cells. Whereas transcriptional activity of Dorsal and Dif has not been previously shown to be regulated by acetylation, p65 acetylation affects NF-κB-dependent transcription in multiple ways. While K310, K314 and K315 acetylation enhances p65 and hence NF-κB transcriptional activity without affecting DNA binding [13], [14], K218 and K221 acetylation increases NF-κB transcriptional activity by inhibiting its removal from DNA by newly synthesized IκBα [13]. In contrast, K122 and K123 acetylation reduces p65 transcriptional activity by decreasing DNA binding in an IκBα-independent manner [16]. Thus, it is possible that specific lysines are targeted in a timely fashion, and that activatory acetylation might prevail during early stages of the transcriptional response, while inhibitory acetylation will be important for terminating NF-κB activity on genes undergoing prolonged transcriptional activation (e.g. *CXCL10*). Supporting this notion, CCT knockdown in mammalian cells decreased *IκBα* and *CXCL2* expression, as assessed 1 h after TNF stimulation while increasing that of *TNF*, *IL8*, *CCL5* and *CXCL10*, as assessed 3 and/or 16 h thereafter (Fig. 2A), suggesting a different impact of acetylation on early- and late-phase of NF-κB transcriptional activity.

p65 acetylation is controlled via opposing effects of HATs and HDACs [6]. Chaperonin CCT is required for the formation of an enzymatically active HDAC3–SMRT complex [42] that can regulate late phase NF-κB activity by reducing p65 DNA binding, leading to termination of NF-κB dependent transcription [13]. This is consistent with the observation that SMRT and CCT co-localize to the promoter region of NF-κB dependent genes [65]. Based on these findings, one would expect CCT knockdown to enhance p65 activity by increasing p65 acetylation, presumably by decreasing the amount of functionally active HDAC3. This is, however, not the case, as CCT knockdown decreased p65 acetylation (Fig. 5A).

Regulation of p65 activity by acetylation is target residue specific. While K310 deacetylation diminishes p65 transcription without affecting DNA binding, decreased K221 acetylation favors IκBα binding, promoting its removal from DNA and nuclear export [13]. Since p65 deacetylation by CCT knockdown promotes NF-κB-regulated transcription, it is unlikely that CCT knockdown is associated with decreased acetylation of these residues during late phase of NF-κB activation. Consistent with this, CCT knockdown increased transcriptional activity of p65 K310R mutant 16 h after TNF stimulation in a similar manner as observed in MEF expressing WT p65 (Fig. 5D). On the other hand, the p65 K221R mutant showed significantly lower transcriptional activity, as assessed 16 h after TNF stimulation (Fig. 5D), possibly reflecting enhanced nuclear export after *de novo* IκBα synthesis [13], and was therefore insensitive to CCT knockdown (Fig. 5D). We cannot, however, exclude that CCT knockdown down-regulates K310 acetylation at earlier time points, which could explain transcriptional repression of *IκBα* and *CXCL2* genes as assessed 1 h after TNF stimulation (Fig. 2A).

Acetylation of p65 K122 and K123 inhibits DNA binding, promoting termination of NF-κB-dependent transcriptional response [16]. In line with this, the p65 K122/123R mutant failed to terminate NF-κB-dependent transcription reflected by increased *Cxcl10* mRNA, as compared to p65 wt or p65 K310R and K221R mutants (Fig. 5D). Interestingly, the kinetics of mRNA expression of this mutant was similar to that observed in CCT depleted cells expressing WT p65, and was insensitive to CCT knockdown (Fig. 5D). This suggests that CCT regulates late phase NF-κB-dependent transcription by enhancing K122 and K123 acetylation.

CBP/p300 is a major p65 acetyltransferase [13], [14], [16], whose catalytic activity is regulated by a multiplicity of factors [66], including auto-acetylation [43]. CBP/p300 has been shown to acetylate p65 K122 and K123 residues [16]. We found that CCT knockdown reduces acetylation of endogenous CBP (Fig. 5B), implicating reduced CBP activity as a mechanism for diminished p65 acetylation and for the failure to terminate NF-κB signaling.

In conclusion this study identifies the chaperonin CCT as a regulator of NF-κB transcriptional activity via a mechanism that targets p65 acetylation, presumably by altering CBP activity. We propose that CCT might be involved in terminating NF-κB signaling and as such may be important for the resolution of inflammation.
